# Robot-Assisted Bimanual Training Improves Hand Function in Patients With Subacute Stroke: A Randomized Controlled Pilot Study

**DOI:** 10.3389/fneur.2022.884261

**Published:** 2022-07-06

**Authors:** Di Ma, Xin Li, Quan Xu, Fei Yang, Yutong Feng, Wenxu Wang, Jian-Jia Huang, Yu-Cheng Pei, Yu Pan

**Affiliations:** ^1^Department of Rehabilitation Medicine, Beijing Tsinghua Changgung Hospital, School of Clinical Medicine, Tsinghua University, Beijing, China; ^2^Department of Physical Medicine and Rehabilitation, Chang Gung Memorial Hospital, Taoyuan City, Taiwan; ^3^School of Medicine, Chang Gung University, Taoyuan, Taiwan; ^4^Graduate School of Science Design Program in Innovation for Smart Medicine, Chang Gung University, Taoyuan, Taiwan; ^5^Center of Vascularized Tissue Allograft, Chang Gung Memorial Hospital at Linkou, Taoyuan, Taiwan

**Keywords:** exoskeleton, neurorehabilitation, robot-assisted bimanual task-oriented therapy, stroke, hand function

## Abstract

**Study Design:**

A randomized controlled pilot study.

**Background:**

Bimanual therapy (BMT) is an effective neurorehabilitation therapy for the upper limb, but its application to the distal upper limb is limited due to methodological difficulties. Therefore, we applied an exoskeleton hand to perform robot-assisted task-oriented bimanual training (RBMT) in patients with stroke.

**Objective:**

To characterize the effectiveness of RBMT in patients with hemiplegic stroke with upper limb motor impairment.

**Interventions:**

A total of 19 patients with subacute stroke (1–6 months from onset) were randomized and allocated to RBMT and conventional therapy (CT) groups. The RBMT and CT groups received 90 min of training/day (RBMT: 60 min RBMT + 30 min CT; CT: 60 min CT for hand functional training + 30 min regular CT), 5 days/week, for 4 weeks (20 sessions during the experimental period).

**Assessments:**

Clinical assessments, including the Fugl–Meyer assessment of the upper extremity (FMA-UE), action research arm test (ARAT), and wolf motor arm function test (WMFT), were conducted before and after the intervention.

**Results:**

Within-group analysis showed a significant improvement in the FMA-UE and WMFT in both the CT and RBMT groups. A significant improvement in the Fugl–Meyer assessment (FMA) of the wrist and hand for the distal part in the RBMT group occurred earlier than that in the CT group. A significant improvement in WMFT time was found in both groups, but the WMFT functional ability assessment was only found in the RBMT group. No significant improvements in ARAT assessment were observed in either the CT or RBMT groups. Compared with CT, significant improvements were found in terms of the proportion of minimally clinically important differences after RBMT in FMA-UE (χ^2^ = 4.34, *p* = 0.037). No adverse events were reported by any of the participants across all sessions.

**Conclusions:**

This study is the first to apply RBMT to the distal part of the upper limb. Both RBMT and CT are effective in improving the upper limb function in patients with subacute stroke. RBMT shows superior potential efficacy in facilitating recovery of the distal part of upper extremity (UE) motor function in the early stage. Future randomized control studies with a large sample size and follow-up assessments are needed to validate the present conclusions.

## Introduction

Every year, 15 million people around the world suffer from stroke (1). More than two-thirds of stroke survivors have impairmed upper extremity (UE) function, and approximately 20% of them have permanently limited hand function (2, 3). Hand function is particularly relevant for activities of daily living; hand dysfunction seriously affects stroke survivors' quality of life and activities of daily life, such as dressing and eating (4–6). Previous studies showed that the recovery of motor function in the upper limb is slower than that in the lower limb in most patients (7, 8). Rehabilitation in the distal part of the upper limb is more challenging than that in the proximal part, due to the high dexterity and degrees of freedom and has a larger coverage in the cortex (9, 10). Task-oriented training, a conventional rehabilitation applied in occupational therapy, is commonly applied to evoke hand function (11), but it is difficult to perform a variety of task-oriented tasks if the motor impairment of a patient in the early stroke stage makes his/her unable to grasp any objects. Thus, it is important to develop effective neurorehabilitation methods to improve the upper limb function.

Bimanual therapy (BMT) is an upper limb neurorehabilitation approach for patients with stroke (12–15) and has demonstrated treatment benefits in improving hand motor function (16) and muscle power (17). In contrast to unilateral training (UMT), neurophysiological studies showed that BMT may involve both the damaged and intact hemispheres, enhancing motor-related brain activity in patients with stroke (18, 19). The mechanism of motor functional restoration induced by BMT involves disinhibition of the motor cortex, which could increase the use of neural pathways circumventing the lesioned brain areas and increase the recruitment of ipsilateral pathways from the contra-lesioned or contralateral brain areas, to supplement the damaged cross-corticospinal pathways and upregulate descending premotor neuron commands to propriospinal neurons (14). The results of meta-analysis studies indicated that both UMT and BMT have comparable treatment effects, but BMT may provide additional benefits in functional improvement (20, 21). However, there are few clinical data because hand disability made it difficult to achieve BMT.

In recent years, robotic-assisted neurorehabilitation has been performed in patients with stroke with hand dysfunction, providing high-intensity, repetitive training (16, 22–26). Robotic devices have also been applied to assist in BMT, in which robotic devices help the movement of the affected limb through active movement of the unaffected UE (23, 27). However, most of these devices are designed for shoulder, elbow, or wrist movements (25, 28), there are few rehabilitation robots for the hand, such as the fingers. For robot-assisted BMT (RBMT) in the hand, Chen et al. and Dong et al. developed a task-oriented training protocol that uses an exoskeleton hand to perform RBMT (29, 30). A functional brain imaging study on RBMT reported that, compared with unilateral hand movement, robot-assisted bilateral hand movement induced greater excitatory responses in the motor cortex (30), suggesting the clinical effectiveness of RBMT.

Thus, in this study, we applied a training program for RBMT in patients with subacute phase of stroke. To achieve RBMT, the robotic device used in the present work is the Mirror Hand, which can control the movement of the affected hand with an exoskeleton hand to mimic the movement of the unaffected hand. Regarding the results from the functional brain imaging in RBMT, we hypothesized that RBMT may contribute to hand functional recovery in patients with stroke.

## Materials and Methods

### Participants

We recruited inpatients with stroke, who had hemiplegic hand function, from the Beijing Tsinghua Changgung Hospital. Inclusion criteria were as follows: (1) first-ever and unilateral ischemic or hemorrhagic cerebrovascular accident diagnosed by computed tomography or magnetic resonance imaging (MRI); (2) patients with subacute stroke with onset between 1 and 6 months; (3) Brunnstrom stages of recovery ranging from 2 to 4; and the (4) modified Ashworth spasticity score of the distal part of the upper limb < 3. Exclusion criteria were as follows: (1) Mini-Mental State Examination score < 24, (2) sensory aphasia or mixed aphasia, (3) hand dysfunction combined with a fracture of the upper limb or hand, and (4) severe neuralgia of the upper limb and hand, affecting training (visual analog scale score > 5). The investigational review board of Beijing Tsinghua Changgung Hospital approved the study protocol, and the participants provided informed consent in accordance with the Declaration of Helsinki (Clinical Trial Registration: ChiCTR1900023989).

### Sample Size and Randomization

The sample size was estimated according to previous studies of BMT and robot-assisted training studies (23, 31, 32), and the sample size was determined to be 10 in both groups. Considering the influence of shedding and elimination (30%), we decided to recruit 30 patients for this pilot. Finally, 32 patients were enrolled. Randomization was mediated through a set of numbered envelopes prepared for each stratum containing cards indicating the allocated group. When a new eligible participant was registered, an envelope was randomly extracted, and the relevant therapist was informed of the group allocation. An investigator was blinded to the treatment, maintained the random sequence, and allocated participants to either RBMT or conventional occupational treatment [conventional therapy (CT)] groups (allocation ratio 1:1). Two certified occupational therapists who were blinded to the group allocation performed the clinical assessments. Each participant was examined pretest and posttest assessments by the same assessor.

### Interventions

Before the study, all occupational therapists (two assessors and 10 therapists) were trained to perform the assessments and rehabilitation procedures to avoid inter-rater scale variability and to ensure consistent treatment by a senior occupational therapist. The RBMT and CT groups received hand training for 90 min/day (RBMT: 60 min RBMT + 30 min regular CT; CT: 60 min CT for hand function + 30 min regular CT), 5 days/week, for 4 weeks (20 sessions during the experimental period). During the study period, all the participants continued to receive regular therapy.

### Exoskeletal Hand Device

The wearable robotic device consisted of an exoskeleton hand, a sensor glove, and a control box (Mirror Hand, HS 001, Rehabotics Medical Technology Corporation). The exoskeleton hand was applied to the patient's affected hand. The exoskeleton hand consists of five individual finger modules, each of which can independently move each of the patient's five fingers on the applied hand. The device provides three motion modes, which are five-finger, individual finger movement mode, and mirror-guided movement mode with a constant speed. Five-finger and individual finger movement modes are performed by the exoskeleton hand alone, which moves five fingers or a single finger to perform extension and flexion passively and continuously. When applying the mirror-guided movement mode, the unaffected hand was applied with the sensor glove, which consists of five sensors, each of which can independently detect the position of each of the patients' fingers in the unaffected hand. The detected signal is transferred to the control box, and then the processed signal is used to control the exoskeleton hand. Mediated by this mechanism, the exoskeleton hand can mirror the movement of the sensory glove and bimanual task-oriented hand training can be achieved.

#### Interventions for the RBMT Group

The program for the RBMT group consisted of 60 min of RBMT training followed by 30 min of regular CT as follows.

The exoskeleton hand was applied to the affected hand, and a sensor glove was placed on the unaffected hand. The participant first received a 5-min five-finger mode continuous passive motion (CPM) exercise at a speed of 15°/s for 6 s, from full extension (0°) to flexion, and then another 6 s back to full extension. The five fingers were moved simultaneously. During CPM training, the patient was asked to move the finger actively or, at least, to move the finger assisted by the movement of the exoskeleton.

The patient then underwent an individual finger CPM exercise. The movement was identical to the five-finger range of motion exercise, except that each finger was sequentially moved in the order of the thumb, index, middle, ring, and pinky fingers, for approximately 3 min.

Then, the patient received RBMT training, in which the patient actively moved the unaffected hand in the sensor glove to control the affected hand on the exoskeleton hand in a mirror-symmetry pattern. Before performing the RBMT program, the patient was instructed to focus mainly on the affected hand and try to move both hands simultaneously.

**Figure 1:**
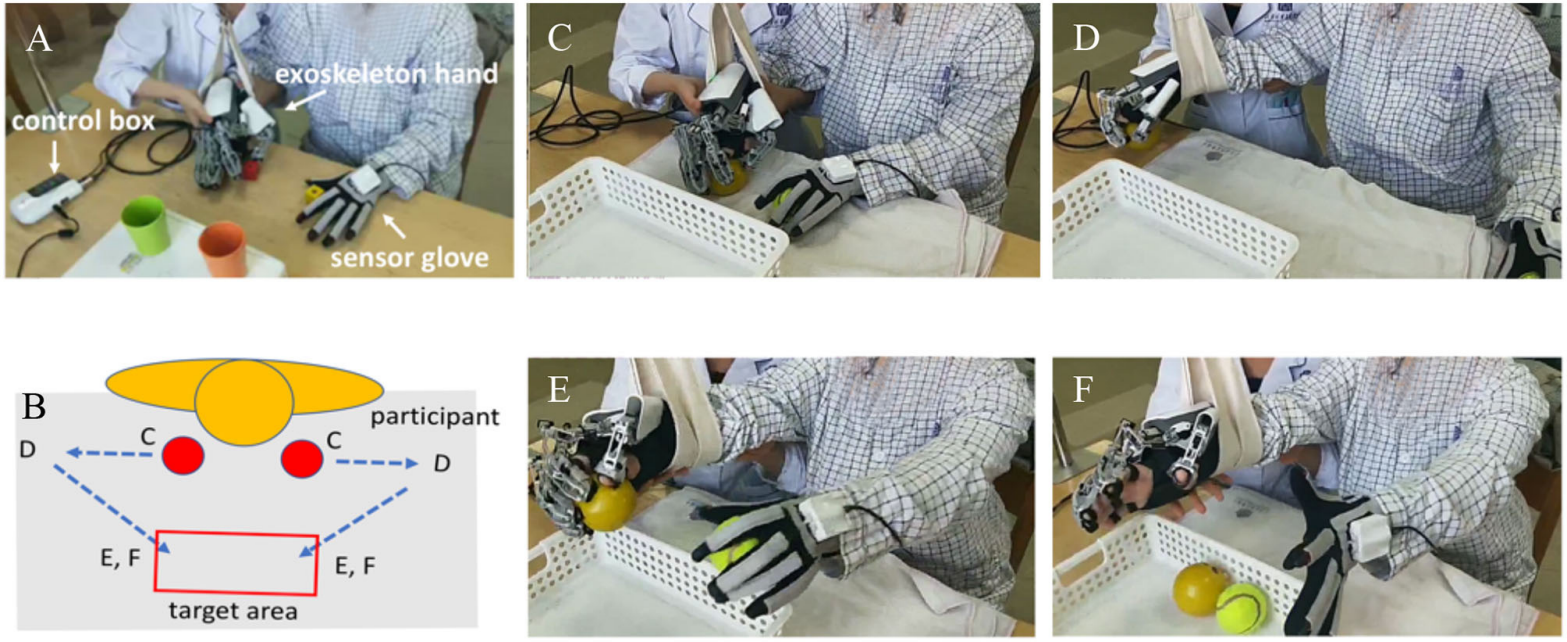
**(A)** The exoskeleton robotic device and **(B)** the motor trace of robot-assisted bimanual therapy (RBMT) used in this study; the circle red was the initial point. **(C–F)** Demonstrated an example of the motor path of a ball gasping/releasing task according to the **(B)**.

Initially, RBMT was conducted without objects (such as grasping, single finger movement, or opposite fingers) for 15 min to make the patient familiar with BMT processes before task-oriented training. Then, the patient was asked to manipulate objects and achieve a specific task with a triangular path, as shown in Figure 1. According to the patient's condition, therapists would select two to three task items: grasping and moving balls, grasping and moving wooden sticks, lifting and moving conical cylinders, pinching and moving wooden blocks, and pinching and moving pegs. Each task item was performed for 10–15 min. In the task-oriented training, a suspension device was provided to assist the paretic arm of those who had difficulty lifting the paretic arm. There was a 2-min break between task items.

After RBMT training, 30 min of regular CT was applied, which consisted of passive stretching, weight-bearing training, pain management, hand manipulation skills, dexterity training, and task-specific activity training.

#### Interventions for the CT Group

Participants in the CT group received a 60-min one-on-one CT for unilateral hand functional training followed by 30 min of a regular CT. The training tasks for object manipulation in the first 60 min were similar to those of the RBMT program, but the task was assisted by a therapist. The following 30 min of regular CT consisted of passive stretching, weight-bearing training, pain management, hand manipulation skills, dexterity training, and task-specific activity training.

### Outcome Measurements

Assessments were performed before treatment (baseline) and after the 10th (*T*_1_) and 20th (*T*_2_) interventions. The clinical assessments applied in this study were as follows.

#### UE Fugl–Meyer Assessment

The Fugl–Meyer assessment for the UE (FMA-UE) is a quantitative assessment tool that measures motor recovery after stroke in the shoulder, elbow, forearm, wrist, and hand. The FMA-UE total score ranged from 0 to 66. It uses a three-point ordinal scale for evaluation (0, cannot perform; 1, perform partially; and 2, perform completely). The minimally clinically important difference (MCID) in the subacute stage for FMA-UE and FMA for the wrist and hand (FMA-WH) was nine and three, respectively (33).

#### Wolf Motor Arm Test

Time-based wolf motor arm function test (WMFT) consists of 17 items with six items for the joint segment (items 1–6), nine items for functional tasks (items 7–15), and two items for strength measurement (34). The performance time of each item was measured between a precisely defined start and end point of a task with a maximum duration of 120 s. The WMFT contains a six-point functional ability scale (WMFT-FAS) that rates movement quality by yielding a score from 0 (no attempt made to use the more affected UE) to 5 (movement appears to be normal). The WMFT-FT (the functional movement items 7–15) was analyzed to evaluate the functional activity (31, 34). The MCID for WMFT is 0.3 for WMFT-FAS and 19 s for WMFT-time (35, 36).

#### Action Research Arm Test

The action research arm test (ARAT) is a performance test that assesses the ability to perform gross motor movements, and grasp, grip, and pinch functions. The original test consists of 19 items rated by four-point ordinal scales. The total score ranged from 0 to 57 points. The MCID of ARAT is set at 12 points (35).

### Statistical Analysis

The data were processed using SPSS (Ver. 25.0, IBM Corp., Armonk, NY, USA). The Jarque–Bera test was used to test the normality of all parameters (α = 5%) (37–40). Nonparametric analyses were applied as the data did not follow a normal distribution. In addition, the sample size is moderate in this study. Demographic and clinical characteristics of the participants were analyzed to evaluate homogeneity between the two groups. Independent *t*-test was used for age, time of stroke onset, FMA-UE, FMA for shoulder and elbow (FMA-SE), FMA-WH, WMFT, WMFT-FT, WMFT-time, and ARAT score. The chi-squared test was applied for categorical variables (sex, stroke type, and side of the lesion). Friedman's test and multiple Wilcoxon signed-rank tests were performed for within-group analysis of the hand functional changes at *T*_2_- and *T*_1_ baseline. Following Bonferroni's adjustment, the significance level in the Wilcoxon signed-rank tests was set at 0.0167. The chi-squared test was used to compare the percentage of participants in whom hand functional recovery exceeded the MCID between the CT and RT groups. All data are presented as mean ± standard deviation (SD).

## Results

**Figure 2:**
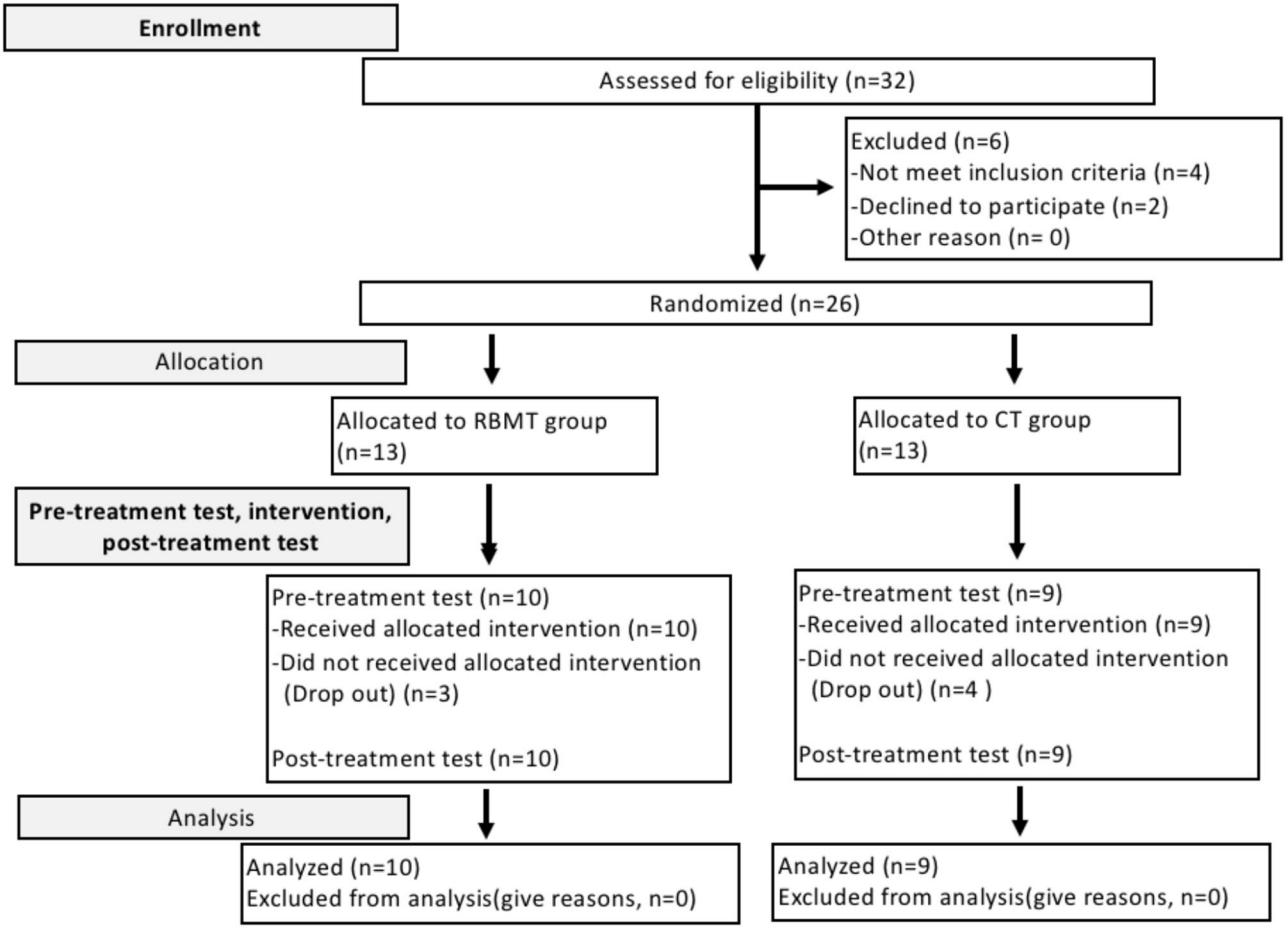
The CONSORT flowchart.

In total, 32 participants were invited to participate in this study. Two were excluded for personal reasons, and four did not meet the inclusion criteria. The remaining 26 patients met our inclusion criteria and were randomly assigned to the CT and RBMT groups (Figure 2). Three patients in the RT group and four in the CT group did not complete the experiment due to personal reasons and hospitalization. Finally, data from 19 participants (CT group: *n* = 9; RBMT group: *n* = 10) were analyzed (Table 1). The analysis showed that the two groups did not differ in any of the parameters in the baseline evaluation, including the FMA-UE, FMA-SE, FMA-WH, WMFT, WMFT-FT, WMFT-time, and ARAT. The stroke onset period was <6 months for all patients, and the severity of upper limb dysfunction was moderate to severe, according to FMA-UE results. No adverse events were reported in this study. FMA-UE and WMFT-time, which reflect the time required to complete the tasks of hand function, also showed improvements at *T*_1_ as compared to the baseline in both the RBMT and CT groups, and the same effects were also observed at *T*_2_.

**Table 1:** The summary of participant's characteristics.

**Variable**	**RBMT group**	**CT group**	***p*-value**
	**(*n* = 10)**	**(*n* = 9)**	
**Sex (** ***n*** **)**			
Male / female	9/1	5/4	0.141^c^
**Age (y)**	59.00 ± 10.60	56.44 ± 8.79	0.577^t^
**Lesion side of brain (n)**			
Right- / left- side	4/6	5/4	0.656^c^
**Stroke type (n)**			
Hemorrhagic / ischemia	0/10	2/7	0.211^c^
**Stroke onset (w)**	10.00 ± 5.85	10.33 ± 6.24	0.906^t^
**FMA-UE score**	27.20 ± 17.03	22.56 ± 17.17	0.562^t^
FMA-SE score	19.90 ± 9.88	15.33 ± 10.61	0.983^t^
FMA-WH score	7.30 ± 8.11	7.22 ± 7.36	0.345^t^
**ARAT score**	10.90 ± 10.33	12.67 ± 14.37	0.760^t^
**WMFT-FAS**	24.20 ± 14.67	23.33 ± 17.80	0.909^t^
**WMFT-FT**	9.50 ± 8.15	10.11 ± 10.06	0.886
**WMFT-time (s)**	71.07 ± 34.41	79.79 ± 38.80	0.610^t^

c *Chi-squared test*;

t *Independent t-test*.

In Table 2, a significant improvement was observed in both the CT and RBMT groups. In the CT group, the effect of improvement in FMA-UE was maintained from *T*_1_ to *T*_2_. The subscores of FMA-SE and FMA-WH only increased at *T*_2_ compared with baseline. In the RBMT group, similar results in FMA and the subscores were found as compared with the CT group. Interestingly, a significant increase in FMA-WH was observed at *T*_1_ in the RBMT group, which is earlier than in the CT group.

**Table 2:** Within-group comparisons of clinical assessment scores.

	**CT group (*****n*** **= 9)**			**RBMT group (*****n*** **= 10)**		
**Variable**	**Baseline**	***T*_1_**	***T*_2_**	**Baseline**	***T*_1_**	***T*_2_**
FMA-UE	22.56 ± 17.17	26.78 ± 18.95*	30.11 ± 20.95^†^	27.20 ± 17.03	31.90 ± 17.01*	36.40 ± 16.87^†^
FMA-SE	15.33 ± 10.61	17.00 ± 11.73	18.67 ± 13.33^†^	19.90 ± 9.88	21.70 ± 9.38	23.00 ± 9.35^†^
FMA-WH	7.22 ± 7.36	9.78 ± 7.74	11.44 ± 7.86^†^	7.30 ± 8.11	10.10 ± 7.98*	13.40 ± 7.73^†^
WMFT-FAS	23.33 ± 17.80	26.33 ± 19.36	30.22 ± 20.02	24.20 ± 14.67	29.80 ± 15.64*	33.60 ± 16.40^†^
WMFT-FT	10.11 ± 10.06	12.78 ± 11.70	15.33 ± 13.27	9.50 ± 8.15	13.60 ± 9.89*	16.20 ± 10.92^†^
WMFT-time(s)	79.79 ± 38.80	70.56 ± 45.28*	64.86 ± 44.47^†^	71.07 ± 34.41	57.25 ± 39.9*	53.05 ± 38.89^†^
ARAT	12.67 ± 14.37	16.56 ± 17.33	18.44 ± 18.17	10.90 ± 10.33	15.50 ± 12.60	20.00 ± 16.51

T_1_ vs. baseline (*); T_2_ vs. baseline (†).

*Significance levels are p < 0.017*.

The assessment of WMFT-time, which reflects the time required to complete the tasks of hand function, also showed improvement at *T*_1_ as compared with baseline in both RBMT and CT groups. The effect of improvement in WMFT-time was also maintained from *T*_1_ to *T*_2_ in both groups. However, the improvement in WMFT-FAS and WMFT-FT observed at *T*_1_ and *T*_2_, which reflects the UE and hand functional task ability, was only found in the RBMT group. In ARAT, there was no significant improvement in any of the two groups.

To compare the magnitude of improvement between the RBMT and CT groups, we applied MCID analysis, which compares the likelihood that patients' improvement had reached clinically noticeable differences at time point *T*_2_. The results showed that the proportion of patients with an MCID in the FMA-UE was higher in the RBMT group than in the CT group (χ^2^ = 4.34, *p* = 0.037, Table 3), supporting the possibility of a greater therapeutic effect of RBMT on upper limb function.

**Table 3:** Comparison of intervention effects to the minimal clinically important difference (MCID) in the Fugl–Meyer assessment of the upper extremity (FMA-UE), wolf motor arm function test (WMFT), WMFT-FAS, and action research arm test (ARAT) assessments.

	**Group**	***T***_**2**_ **- baseline**		
		**MCID**	**Mean**	**Percentage**
FMA-UE	CT	9	7.56	22.22% (2/9)
	RBMT		9.20	70% (7/10)^*^
WMFT-FAS	CT	0.3	0.46	66.67% (6/9)
	RBMT		0.63	90.00% (9/10)
WMFT-time (s)	CT	9	14.93	33.33% (3/9)
	RBMT		18.02	40.00% (4/10)
ARAT	CT	12	9.10	22.22% (2/9)
	RBMT		5.78	40.00% (4/10)

* *p < 0.05, Chi-squared test*.

Finally, to explore whether treatment effects differed in patients with different severities of hand function impairment, we performed a subgroup analysis by assigning patients to two severity subgroups, the moderate (FMA-UE ≥ 20) and severe (FMA-UE < 20) severity subgroups according to the cutoff point proposed by Michaelsen et al. (41), and examined whether the aforementioned improvement of FMA-UE was found in both subgroups. The results showed that, in the severe severity subgroup, the increase in FMA-UE in the RBMT group was higher than that in the CT group (FMA-UE, *T*_2_-baseline, CT group = 4.00 ± 2.92, RBMT group = 9.40 ± 2.61, *Z* = 2.312, *p* = 0.016, *r*^2^ = 0.731), but this effect was not observed in the moderate severity subgroups. This difference in improvement between the two severity subgroups was not observed in other assessments.

## Discussion

In the present pilot study, our results demonstrated significant increases in hand function induced by CT and RBMT interventions for patients with subacute stroke. RBMT showed additional benefits in FMA-WH and WMFT-FT compared with CT. In a subgroup analysis of severity according to the FMA-UE classification, a significant functional improvement was found in patients with severe UE dysfunction (FMA-UE score <20) in the RBMT group. Due to the limitation of small sample size and follow-up assessments, further studies are needed to validate our preliminary findings. No adverse events were reported by any of the participants across all sessions.

The MCID results showed that functional improvement on the FMA-UE was more pronounced in the RBMT group. Stroke-induced functional recovery is more likely to be captured by FMA-UE scores, as this scale focuses on the motor activity of the upper limbs. Indeed, the FMA-UE was reported as a widely used clinical scale to detect motor recovery of upper limb functions and is considered a suitable tool to measure the effect of neurorehabilitation (42). Better improvement of FMA-UE in the RBMT group might be explained by two factors. First, high-dose, focused arm and hand therapy, and better postural control, appear to promote neurophysiological recovery after stroke. Second, RBMT focuses on improving hand impairment and, therefore, elicits greater benefits in reducing compensatory trunk movements at the start of reaching (25).

In the present work, neither group improved in the ARAT assessment. The reason may be that the functional disability in the participants we enrolled was severe, and a longer treatment period may be needed at this stage. Although both groups showed a significant improvement in UE motor function in FME-UE and WMFT, the improvement may be insufficient to yield a significant improvement in ARAT assessment. The other reason is that the WMFT assessment allows the unaffected hand to move the affected hand to complete the task, whereas the ARAT task is asked to be performed unilaterally, making it difficult to perform.

The current study found that RBMT is superior in improving the ability to execute functional movements and performance of activities, as compared with CT. This finding may be related to neuroplasticity, which can further promote the acquisition of motor skills by RBMT. No previous study has used robotic-assisted, task-oriented BMT while a patient with stroke manipulated real objects. The patient could manipulate objects of specific shapes, sizes, and materials, using his/her affected hand, which may involve sensory feedback from the hand to the central nervous system, participating in neuroplasticity changes in the injured brain. The literature indicates that undamaged neural function in the injured or contralateral brain areas can be involved in the creation of premotor neuron commands (14). This may be the reason for hand functional improvements in both treatment groups in this study. However, as the percentage of FMA-UE improvements was higher in the RBMT group than in the CT group, the improvement in the WMFT-FAS and WMFT-FT was only observed in the RBMT group. The results suggest that the application of an exoskeleton to assist hand training can facilitate the recovery of UE and hand functional performance.

The lack of significant differences in the assessments of FMA-UE, ARAT, WMFT-time, and WMFT-FAS between the CT and RBMT groups in this study could apply to a small sample size. In addition, studies have shown that the subacute phase of stroke represents a potential window for recovery. Indeed, our findings were analogous to those of a study that applied robot-assisted rehabilitation of hand function in the subacute phase of stroke (43) and found that the therapy and control groups yielded comparable improvement. Meyer et al. (32) indicated that early delivery of the arm-hand focused boost program was superior to delayed therapy for motor performance of FMA-UE. Thus, training programs, such as RBMT or control training, that applied highly intensive and specific task training for patients with stroke during the early recovery stage could produce clinically meaningful treatment effects.

A significant improvement of UE function in patients with severe UE dysfunction (initial FMA < 20) after RBMT training may suggest that patients with severe UE dysfunction may benefit more from RBMT than those with mild to moderate dysfunction. Ranzani et al. performed a randomized controlled trial involving mainly mildly or moderately impaired (FMA-UE score > 29) patients with subacute stroke and found that motor recovery in the robot-assisted group was not inferior to that in the CT (43), suggesting that patients with minor deficits might have a ceiling effect on motor recovery; thus, the effect of upper limb training was masked (44). Within this perspective, RBMT may serve as a complementary treatment in the early rehabilitation of stroke survivors, especially for those with severe UE dysfunction.

The present pilot study has several limitations. First, the main limitation of this study was a small sample size; a large sample size will be needed in the future to further validate the treatment effect. The WMFT-FAS can be used to reflect the performance of hand functional movement. Therefore, we estimated the acceptable sample size according to the present pilot study, we suggested that the sample size for an RCT study in the future would be around 42 patients in each group (α = 0.05, and a power of 0.8). Second, we could not conduct follow-up assessments to analyze the retention of training effects because most of the participants were unwilling to come back after discharge due to the long distance from our hospital, while registered therapists in our region were restricted from home visits because the policy reasons. Another important reason is the start of the COVID-19 pandemic in China.

## Conclusion

To the best of our knowledge, this pilot study is the first to reveal the therapeutic effect of robot-assisted bimanual training on the distal part of the upper limb in patients with stroke. Both RBMT and CT are effective in improving the upper limb function of patients with subacute stroke. RBMT shows superior potential efficacy in facilitating recovery the motor function of the distal part of UE in the early stage. Future randomized control studies with large sample size and follow-up assessments are needed to validate the present findings.

## Data Availability Statement

The raw data supporting the conclusions of this article will be made available by the authors, without undue reservation.

## Ethics Statement

The studies involving human participants were reviewed and approved by the Investigational Review Board of Beijing Tsinghua Changung Hospital. The patients/participants provided their written informed consent to participate in this study.

## Author Contributions

DM, J-JH, Y-CP, and YP designed the experiment. DM, XL, QX, FY, YF, WW, and YP participated in the enrollment phase and carried out the treatment procedures. DM, XL, and YP analyzed the data. DM and YP wrote the manuscript. DM, QX, J-JH, Y-CP, and YP interpreted the data. All authors contributed to the article and approved the submitted version.

## Funding

This study was supported by Tsinghua University Precision Medicine Research Program (No. 10001020124), the Capital Health Research and Development of Special (No. 12021B2005), and Beijing Tsinghua Changgung Hospital Youth Start Fund (No. 12019C1008).

## Conflict of Interest

J-JH is the designer of the Mirror Hand and the funder of Rehabotics Medical Technology, Taiwan. The remaining authors declare that the research was conducted in the absence of any commercial or financial relationships that could be construed as a potential conflict of interest.

## Publisher's Note

All claims expressed in this article are solely those of the authors and do not necessarily represent those of their affiliated organizations, or those of the publisher, the editors and the reviewers. Any product that may be evaluated in this article, or claim that may be made by its manufacturer, is not guaranteed or endorsed by the publisher.
